# Effectiveness of Clinical Decision Tools in Predicting Pulmonary Embolism

**DOI:** 10.1155/2021/8880893

**Published:** 2021-02-19

**Authors:** Michael A. Simon, Christopher Tan, Patrick Hilden, Lyle Gesner, Barry Julius

**Affiliations:** ^1^Department of Radiology, RWJBH–Saint Barnabas Medical Center, Livingston, New Jersey, USA; ^2^Department of Biostatistics, RWJBH–Saint Barnabas Medical Center, Livingston, New Jersey, USA

## Abstract

**Objective:**

The Wells criteria and revised Geneva score are two commonly used clinical decision tools (CDTs) developed to assist physicians in determining when computed tomographic angiograms (CTAs) should be performed to evaluate the high index of suspicion for pulmonary embolism (PE). Studies have shown varied accuracy in these CDTs in identifying PE, and we sought to determine their accuracy within our patient population.

**Methods:**

Patients admitted to the Emergency Department (ED) who received a CTA for suspected PE from 2019 Jun 1 to 2019 Aug 31 were identified. Two CDTSs, the Wells criteria and revised Geneva score, were calculated based on data available prior to CTA and using the common D-Dimer cutoff of >500 *μ*g/L. We determined the association between confirmed PE and CDT values and determined the association between the D-Dimer result and PE.

**Results:**

392 CTAs were identified with 48 (12.1%) positive PE cases. The Wells criteria and revised Geneva score were significantly associated with PE but failed to identify 12.5% and 70.4% of positive PE cases, respectively. Within our cohort, a D-Dimer cutoff of >300 *μ*g/L was significantly associated with PE and captured 95.2% of PE cases.

**Conclusions:**

Both CDTs were significantly associated with PE but failed to identify PE in a significant number of cases, particularly the revised Geneva score. Alternative D-Dimer cutoffs may provide better accuracy in identifying PE cases.

## 1. Introduction

Approximately 180,000 people die each year from pulmonary embolism [1]. Chest computed tomographic angiograms (CTA) are frequently used in the Emergency Department (ED) setting to rule out PE [1]. To guide physicians in managing patients with suspected PE and determine the appropriateness of a CTA, two well-known clinical decision tools (CDTs), the Wells criteria and the revised Geneva score, have been developed [2, 3]. The Wells criteria and the revised Geneva score both stratify patients into risk groups based on clinical characteristics and combine these risk groups with the results of a D-Dimer test in order to determine a patient's likelihood of PE and subsequent need for a CTA (Tables 1 and 2).

The Wells criteria have two accepted methods for calculating PE risk and guidance for a CTA, the two-tiered and three-tiered models [2, 4]. The criteria for assigning points based on signs/symptoms remain the same between these models; the stratification of patients into their risk category for PE differs, which in turn changes the recommended action. The three-tiered model separates the patients into low, medium, and high risk which represents patients who scored 0-1, 2-6, and ≥7 points, respectively. According to this model, patients with a high risk score should receive a CTA while those who fall under low or intermediate risk should receive either a standard D-Dimer using the ELISA method or a high sensitivity D-Dimer test using fluorescent immunoarrays. If the D-Dimer is positive, these patients should receive a CTA, whereas the two-tiered model states that patients with scores ≤ 4 are considered PE unlikely and should receive a high sensitivity D-Dimer test. If positive, a CTA should be performed. Patients with scores > 4 are considered PE likely and should subsequently undergo a CTA [2, 4].

Although these clinical decision tools are available to guide physicians on whether or not a CTA is warranted, many continue to rely on personal experience with several studies highlighting that CDTs are underutilized [1, 5]. The underutilization of CDTs has been attributed to the experience of the treating physician, a lack of trust in CDTs, and concerns about the impact of false-negative findings [6]. However, studies have shown that most clinicians make errors in diagnosis when faced with complex cases, with up to 35% of these mistakes resulting in morbidity [6, 7].

While CTA remains the gold standard for PE evaluation, CTAs put patients at additional risks for malignancy due to high radiation dose targeted to the chest and renal failure due to the usage of iodine contrast. Additionally, given the finite availability of CT time in the emergency setting, CTA should only be utilized when necessary [8–10].

## 2. Methods

Institutional IRB approval with a waiver for informed consent was obtained for this retrospective study. A chart review was performed between June 1^st^ through August 31^st^, 2019 (three months immediately prior to the IRB approval and represented approximately 400 cases) and identified CTAs ordered for patients located in the ED with concern for PE. Review of the medical records did not reveal any documented Wells criteria or revised Geneva score by the ED physicians. Therefore, clinical data, i.e., variables for the respective scoring systems, were extracted from the electronic medical record with subsequent calculation of the Wells criteria and revised Geneva score. Only data available to the ordering physician at the time of the patient's CTA was included in the score calculation.

We utilized the two-tiered model of the Wells criteria, which uses a cutoff of ≤4 and >4. This was done to minimize the subjective aspect of the Wells criteria. When calculating the Wells criterion score, the criterion “PE likely or more likely than an alternative diagnosis” was considered positive only if explicit concern was documented in the ED note. When calculating the revised Geneva score, the first recorded heart rate on presentation was used. In line with previous studies, we combined the intermediate- and high-risk groups together and used the cutoff of ≤3 to effectively rule out PE, thus separating the revised Geneva score into two groups with low or intermediate/high risk, separated by their need for further testing [11, 12].

Clinical findings of deep vein thrombosis (DVT) were considered positive based on a physical exam and if ultrasound of the lower extremity demonstrated a DVT. In addition, a high sensitivity D-Dimer value of greater than 500 *μ*g/L was considered positive [13].

An analysis based on the respective CDT algorithms utilizing both the CDT and D-Dimer values was performed. Each CDT was separated based on whether a CTA would have been indicated based on the results of the CDT. The association between PE, patient, and treatment characteristics, and each CDT was assessed using the Wilcoxon rank-sum test for continuous variables and the chi-squared or Fisher's exact test for categorical variables as appropriate. ROC analyses were used to determine the benefit of other potential cutoffs for D-Dimer testing within our population. All analyses were completed in R (version 3.6.2).

## 3. Results

A total of 392 cases (ED admits) were identified among 376 unique patients; 48 (12.1%) of cases were found to have PE on CTA. 266 (67.9%) of cases were female, and the median (range) age of the study population was 55 (17–97) years. The median Wells score was 1 (0–9) with thirty-eight cases (9.7%) having a score greater than 4. The median revised Geneva score was 4 (0, 12) with 224 (57.1%) of cases having a revised Geneva score greater than 3. D-Dimer values were available for 155 cases (39.5%) with 61 (39.4%) being >500 *μ*g/L.

When analyzing our data according to the accepted algorithms of the Wells criteria and revised Geneva score, both the Wells criteria and revised Geneva score were significantly associated with PE (*p* < 0.001 and *p* = 0.033, respectively) (Table 3). Among patients positive for PE, the Wells criteria appropriately guided the physician in 88% of cases to order a CTA (true positive), while among patients negative for PE, 41% were guided to CTA (false positive). Within the revised Geneva score, only 30% of patients with PE received a CTA correctly while 12% of negative PE patients received a CTA.

Among the subset of 155 cases with a D-Dimer, we identified a value of >300 *μ*g/L as being significantly associated with PE (*p* = 0.021) and accurately captured 20/21 (95.2%) positive PE cases among this subset (Tables 4 and 3). As for the 206 patients that did not have a D-Dimer performed and had Wells criteria < 4, there were sixteen cases positive for PE, resulting in a positivity rate of 7.7%. In comparison, our analyzed cohort which had D-Dimers available but Wells criteria < 4, the positivity rate was 10.1%. This difference in positivity rate is suggestive that analyzing by gestalt alone is creating overuse.

## 4. Discussion

When the Wells criterion algorithm was followed utilizing the accepted cutoff of 500 *μ*g/L, the Wells criteria were significantly associated with CTA result accurately directing 28/32 PE cases to subsequent CTA. Although the Wells criteria were significantly associated with CTA, 12.5% of patients would have been missed suggesting that this scoring method and cutoff may not be ideal for our patient population. In addition, the Wells criteria are heavily weighted towards physician evaluation of the patient and are thereby a more subjective scoring system. This could lead to an increased amount of CTAs ordered based on physician experience. The revised Geneva score did not provide a clinically viable alternative scoring system, with 19/27 PE cases not directed to CTA. Similar results have been reported which found the revised Geneva score to be less robust than the Wells score in predicting PE [11, 14].

Our findings reiterate results that have been seen in other studies which describe a modest 10% positive rate of CTAs for PE while using a CDT. This suggests not only a general overuse of CTAs but an inadequacy of our current CDTs [6, 15]. The failure of the current CDTs to predict a positive test could be due to the tools being built on specific patient populations which do not extrapolate to other groups. Therefore, prior to use, a CDT may need to be evaluated for the specific population to ensure its validity.

This has been done in other institutions such as Allegheny General Hospital in Pittsburgh, Pennsylvania, where a study suggested that, although a value of 500 *μ*g/L is accepted as the D-Dimer cutoff, different values could provide for better care. For instance, raising the threshold to 850 *μ*g/L kept the sensitivity at 100% while increasing specificity to 51% in their population [16]. Further analysis of our data revealed that a value of >300 *μ*g/L was significantly associated with positive CTA. Using this cutoff value as opposed to 500 *μ*g/L greatly increases our sensitivity while slightly decreasing the specificity, missing only one positive PE case (4.7% of cases). This new cutoff value in combination with the Wells criteria could potentially reduce the amount of CTAs performed while maintaining an acceptable level of sensitivity. Our data also suggested that the use of D-Dimer alone may be able to predict CTA outcome just as well as a combination of D-Dimer and a CDT.

CTAs are not benign tests. Carcinogenic risks from radiation delivered during imaging studies are often debated. The variability of radiation dose from CT scans and the uncertain effect of the carcinogenic risk from low dose radiation (0-100 mSv) further confound the matter [17]. However, analysis of atomic bomb survivors (long and accurate follow-up) suggests that there is an increased risk of malignancy with radiation doses between 5 and 100 mSv [17]. Further, studies have shown that there is a higher risk of breast cancer 10 years following two CT chest exams than in a female who did not receive any CT exams during the same period [18]. Alterations to the scoring systems, especially for our predominantly female population, to better stratify patients must be determined.

## 5. Conclusion

Although CDTs may be useful, our data has exposed concerns in the applicability of the Wells and revised Geneva scores within our population. The data we collected suggests that the revised Geneva score is not applicable to our patient population. The Wells criteria, although associated with PE, missed 12.5% of positive cases. This would suggest that further analysis of the Wells criteria is warranted in our patient population. Identifying a better D-Dimer cutoff could be beneficial in reducing scans. Association of D-Dimer alone in comparison to D-Dimer with the Wells criteria is also warranted based on our findings. Based on this, we are currently performing a prospective study to identify an ideal cutoff for D-Dimer in our patient population. The overall low positive rate of CTA with the use of CDTs suggests the need for further refinement of these tests [6, 15].

Furthermore, comparing the lower positivity rates of the low-risk, non-D-Dimer-performed cohort (7.7%) with the low-risk, D-Dimer-performed cohort (10.1%), it is suggested that determining the need for a low-risk patient to undergo a CTA using gestalt instead of using a D-Dimer is less accurate and contributes to overuse.

## 6. Limitations

This is a retrospective study at a single institution with a limited sample size. Additionally, a misclassification bias may be present in our analysis as the ED physicians did not document the Wells criteria or revised Geneva in the patient's charts. Therefore, assumptions were made when calculating the Wells criteria, such as only adding three points if the concern for PE was expressly documented. This method could have artificially lowered the Wells criteria for multiple patients placing them in a category where a CTA was not needed. Both clinical decision tools rely on D-Dimer testing in conjunction with the scoring system. More than 230 patients from our cohort did not have a D-Dimer performed. Of these patients, 27 had a positive CTA. The lack of D-Dimer testing prevented our team from fully evaluating the usefulness of the scoring systems for evaluation of PE. Our prospective study will include a D-Dimer sample from all patients.

## Figures and Tables

**Table 1:** Wells criteria.

Variable	Score
Clinical signs and symptoms of DVT	3
PE is #1 diagnosis or equally likely	3
*Heart* *rate* > 100	1.5
Immobilization^a^ or surgery^b^	1.5
Prior, objectively diagnosed PE or DVT	1.5
Hemoptysis	1
Malignancy^c^	1

^a^At least three days; ^b^in previous four weeks; ^c^treatment within 6 months or palliative care.

**Table 2:** Revised Geneva score.

Variable	Score
*Age* > 65	1
Prior DVT or PE	3
Surgery^a^ or lower limb fracture in past month	2
Active malignant condition^b^	2
Unilateral lower limb pain	3
Hemoptysis	2
Heart rate	
<75	0
75-94	3
≥95	5
Pain on lower limb palpation and unilateral edema	4

^a^Under general anesthesia; ^b^solid or hematologic malignant condition currently active or considered cured <1 year.

**Table 3:** Wells criteria, revised Geneva score, and D-Dimer association with CTA. Median (range), *N* (%).

	CTA result		
Variable	Positive	Negative	*p* value
Wells criteria	1.5 (0.0, 9.0)	0.0 (0.0, 7.5)	<0.001
Wells criteria (D-Dimer cutoff 500)			<0.001
CTA	28 (87.5)	63 (41.2)	
No CTA	4 (12.5)	90 (58.8)	
Missing	16	191	
Revised Geneva score	5.5 (0.0, 12.0)	4.0 (0.0, 12.0)	0.02
Revised Geneva score (D-Dimer cutoff 500)			0.033
CTA	8 (29.6)	25 (12.0)	
No CTA	19 (70.4)	183 (88.0)	
Missing	21	136	
D-Dimer	1066.0 (273.0, 15180.0)	394.0 (150.0, 8653.0)	<0.001
D-Dimer			0.046
≤300	1 (4.8)	32 (25.8)	
>300	20 (95.2)	92 (74.2)	
Missing	27	220	

**Table 4:** Demographics, Wells criteria, revised Geneva score, and D-Dimer median (range), *N* (%).

Variable	Summary
Gender	
Male	126 (32.1)
Female	266 (67.9)
Age at CTA	55 (17, 97)
CTA	
Negative	344 (87.8)
Positive	48 (12.2)
Wells criteria	1 (0, 9)
Revised Geneva score	4 (0, 12)
Wells criteria (D-Dimer cutoff 500)	
CTA	91 (49.2)
No CTA	94 (50.8)
Missing	207
Revised Geneva score (D-Dimer cutoff 500)	
CTA	33 (14.0)
No CTA	202 (86.0)
Missing	157
D-Dimer	421 (150, 15180)
≤300	33 (22.8)
>300	112 (77.2)
Missing	247

Cases were considered missing when the data was not complete, i.e., no D-Dimer.

## Data Availability

The data used to support the findings of this study are included within the article. Additional data maybe available upon request from the institutional IRB.

## Additional Points

*Take Home Points*. (1) Our data suggests that the revised Geneva score fails to capture a significant proportion of patients with pulmonary embolism. (2) The Wells criteria were significantly associated with pulmonary embolism when using a D-Dimer cutoff of 500 *μ*g/L; however, the scoring criteria still missed 13% of cases. (3) A D-Dimer cutoff of ≥300 accurately captured 20/21 positive cases (95% sensitivity and 25% specificity), suggesting that the D-Dimer itself may be an adequate screening tool for PE. Further investigation of D-Dimer alone or in conjunction with the Wells criteria is warranted.
